# Smartphone application‐delivered cognitive behavioural therapy for insomnia with telephone support for insomnia disorder compared to a waitlist control: a randomised clinical trial

**DOI:** 10.1111/jsr.14363

**Published:** 2024-10-08

**Authors:** Markus Jansson‐Fröjmark, Rikard Sunnhed

**Affiliations:** ^1^ Centre for Psychiatry Research, Department of Clinical Neuroscience Karolinska Institutet, and Stockholm Health Care Services Stockholm Sweden

**Keywords:** cognitive behavioural therapy for insomnia, insomnia, smartphone application‐delivered, telephone support

## Abstract

Although there have been promising findings for smartphone application (app)‐delivered cognitive behavioural therapy for insomnia (CBT‐I), previous trials have not screened participants rigorously for insomnia disorder and used therapist support. Based on the above, we aimed to examine the effects of smartphone app‐delivered CBT‐I with telephone support against a waitlist (WL) in a sample with insomnia disorder. A total of 64 participants with insomnia disorder were randomised to smartphone app‐delivered CBT‐I (*n* = 32) or a WL (*n* = 32). Smartphone app‐delivered CBT‐I consisted of six weekly smartphone app modules with 15 min of telephone support per week. At pre‐ and post‐treatment, and the 3‐month follow‐up, we assessed insomnia symptoms and associated correlates and consequences. At post‐treatment, we also assessed measures related to adherence (therapist support, exercise/module completion), self‐rated perception of treatment content, activity, and adverse events. CBT‐I significantly outperformed the WL with large effects on the primary outcome (*d =* 2.26) and was significantly different on most of the secondary outcomes with medium to large effects. CBT‐I also resulted in a significantly larger proportion of treatment remitters (CBT‐I: 64.5–77.4%, WL: 6.5–6.9%) and responders (CBT‐I: 77.4–90.3%, WL: 19.4–24.1%) at post‐treatment and follow‐up, compared to the WL. Treatment was associated with high satisfaction, high adherence, low attrition, and few treatment‐impeding adverse events. Based on the medium to large effects of smartphone app‐delivered CBT‐I with telephone support, this trial highlights the potential of delivering CBT‐I exclusively through an app with therapist telephone support for high efficacy, satisfaction, and adherence.

## 1 INTRODUCTION

Insomnia disorder is defined as having problems with initiating and/or maintaining sleep for at least 3 nights a week, during a minimum of 3 months, with associated significant impairment or distress (Diagnostic and Statistical Manual of Mental Disorders, 5th ed., 2013). Insomnia disorder is considered the most prevalent of all sleep disorders (Ohayon & Reynolds, 2009) and the second most common mental disorder in Europe (Wittchen et al., 2011), with a point prevalence of ~10% (Morin et al., 2015). Insomnia is associated with several negative consequences, such as a higher frequency of work absenteeism, sick leave, and accidents, as well as the risk of developing other physical or mental health problems (Baglioni et al., 2019; Hertenstein et al., 2019). Based on the above, there exists a dire need for accessible, effective, and user‐friendly interventions for alleviating insomnia in the large number of affected individuals.

Cognitive behavioural therapy for insomnia (CBT‐I) is a psychological therapy of short duration that consists of cognitive and behavioural techniques aimed at reversing sleep‐related cognitive and behavioural processes (e.g., irregular sleep patterns, worry, and unhelpful beliefs) proposed to perpetuate insomnia (Edinger & Carney, 2014; Morin & Espie, 2004). Despite CBT‐I being the recommended first‐line treatment, insomnia is still more frequently treated by sleep medications (Baglioni et al., 2019; Ellis et al., 2023). Common reasons why CBT‐I is not used are likely related to several contextual barriers, such as availability and the ease of disseminating pharmacological agents in relation to CBT‐I, where certified and geographically distributed therapists are scarce, the short‐term cost for CBT‐I higher (Baglioni et al., 2019; Thomas et al., 2016), and CBT‐I demanding of more time and energy from both patients and the healthcare providers (Koffel et al., 2018; Riemann et al., 2022). In all, this makes the threshold for disseminating CBT‐I higher than for pharmacotherapy (Koffel et al., 2018; Riemann et al., 2022). One solution to decrease these barriers has been achieved with internet‐delivered CBT, which makes treatment easier to disseminate to a larger part of the population with a lower workload for the therapist. However, many of the available internet interventions are still characterised by several patient barriers that prevent patients from using CBT‐I, such as finding and allocating time between work, social and family leisure, or finding energy for mentally engaging with the treatment content (Kathol & Arnedt, 2016; Koffel et al., 2018).

By instead disseminating CBT‐I digitally through a smartphone application (app), several of these barriers could potentially be diminished. Besides allowing the patients to access and work on treatment from wherever they are and whenever the time is available (e.g., when commuting or during spare time), the app‐format also allows the possibility to deliver screening, assessment, and treatment in one format where all content is placed: sleep‐diaries, questionnaires, psychoeducation (e.g., video animations), exercises, supplementary information, feedback, reminders, and therapist support. Thus, an app could provide an all‐in‐one solution where both therapist and patient can find all they need to assess, monitor, and manage treatment progression. Therefore, smartphone app‐delivered CBT‐I may be one potential route to further close the dissemination gap between CBT‐I and prescription medications (Baglioni et al., 2019; Ellis et al., 2023; Riemann et al., 2022).

To our knowledge, there are 12 studies examining the effect of CBT‐I delivered exclusively through a smartphone app available. Of these, three are of a non‐randomised or open design (Jernelöv et al., 2023; Philip et al., 2022; Stenberg et al., 2022). The remaining nine are randomised controlled trials (RCT) that, compared to passive or active control, also indicate promising results with reductions in primarily insomnia severity as outcome (Chung et al., 2024; Hinterberger et al., 2024; Horsch et al., 2017; Kuhn et al., 2021; Okajima et al., 2020; Reilly et al., 2021; Shimamoto et al., 2022; Watanabe et al., 2022; Zhang et al., 2023). Although all nine RCTs are relevant contributions to understanding the effect of CBT‐I delivered exclusively through a smartphone app, some limitations prohibit a more thorough understanding of the potential benefits of smartphone app‐delivered CBT‐I. For example, none of the previous trials have applied screening procedures that sufficiently capture expert recommendations for a standard research assessment of insomnia (Buysse et al., 2006; Diagnostic and Statistical Manual of Mental Disorders, 5th ed., 2013; Edinger et al., 2004; Lichstein et al., 2003), which makes conclusions on the effect on insomnia disorder limited. Furthermore, besides outcome on insomnia severity, few trials have assessed the controlled effect on broad insomnia‐related constructs, such as night‐time symptoms, functional impairment, quality of life, stress, depression, and anxiety. Moreover, little is known about how the dissemination of CBT‐I through an app is received, such as how patients adhere to the app format and the degree of dropout, factors important for improvement (Horsch et al., 2015; Soh et al., 2020; Trockel et al., 2014; Wickwire, 2019; Zachariae et al., 2016). Furthermore, very little is known regarding measures on participants’ perception of usability, workload, and satisfaction with app‐delivered CBT‐I (e.g., invested time and work, amount of information digested, credibility/expectancy, and relevant support). Finally, none of the previous trials were delivered with support from a human therapist (i.e., using a chat function or a telephone for problem‐solving and support). As meta‐analytic findings have shown superior outcomes for therapeutic guidance compared to non‐human guidance, on both symptom severity and adherence (Baumeister et al., 2014; Hasan et al., 2021), the role of therapist support in app‐based CBT‐I is an interesting, yet unaddressed gap in research.

Based on the abovementioned gaps in knowledge, this study aimed to examine the effect of CBT‐I delivered exclusively through a smartphone app with therapist support, compared to a sleep‐diary waitlist control (WL). The choice of waitlist as a comparator was deemed as a relevant first step, given the uncertainty of whether smartphone app‐based telephone‐supported CBT‐I reduces insomnia in a rigorously screened sample for insomnia disorder. To get a broad picture of this relatively novel way of delivery, the controlled effect of CBT‐I was assessed on insomnia severity, night‐time symptoms, and insomnia‐related problems (i.e., functional impairment, quality of life, stress, anxiety, and depression). Furthermore, the CBT‐I group was assessed on treatment‐relevant factors, such as activity, adherence, dropout, expectancy/credibility, perception of treatment and its support, and adverse events. Based on previous evidence for internet and mobile‐delivered CBT, we hypothesised that smartphone app‐delivered CBT‐I with telephone support would outperform the WL on the primary outcome (insomnia severity). Due to the lack of assessing multiple other outcomes in previous research using app‐delivered CBT‐I, hypotheses for the secondary outcomes were not formulated.

## 2 METHODS

### 2.1 Study design and conditions

This study is reported following the Consolidated Standards of Reporting Trials (CONSORT) guidelines for RCTs (Schulz et al., 2010) and registered as a RCT at clinicaltrials.gov, with approval number: NCT05065242. The trial was conducted as a collaboration between a university in Sweden and the company (Learning to Sleep L2S AB, https://www.learningtosleep.se) responsible for producing and delivering the treatment. The study included 64 participants randomised to either CBT‐I (*n* = 32) or a WL control group (*n* = 32). The number of participants included was based on a priori analyses using the GLIMMPSE online power computation for linear models (Kreidler et al., 2013). The power analysis was specified for detecting a significant change from pretreatment to the 3‐month follow‐up with a power of 80% and an alpha of 0.01 for the primary outcome. The model for estimated power was based on the observed mean reduction from pretreatment to the 3‐month follow‐up in the Insomnia Severity Index (ISI) (ISI score CBT‐I: 16.6 to 11.0, WL: 16.7 to 15.8) in two recent trials of smartphone app‐based CBT‐I (Horsch et al., 2017; Kuhn et al., 2021), and specified as the linear change from pretreatment to the 3‐month follow‐up using seven assessment points (Weeks 0, 1, 2, 3, 4, 5, and 18), and an expected study dropout of 24.7% (Zachariae et al., 2016). The inclusion of 64, in contrast to the pre‐registered 90, was due to a renewed power analysis that, as mentioned above, made use of all seven measurement points, which, due to its interaction with the number of participants, requires a smaller sample size for reaching the same specified power.

The study received ethical approval by the Regional Ethical Board in Stockholm, Sweden (reference number 2021–01347). Participants gave informed consent as a written digital signature, and data were handled through a secure online platform that ensured anonymity and safety during the whole treatment period.

### 2.2 Participants

**FIGURE 1:**
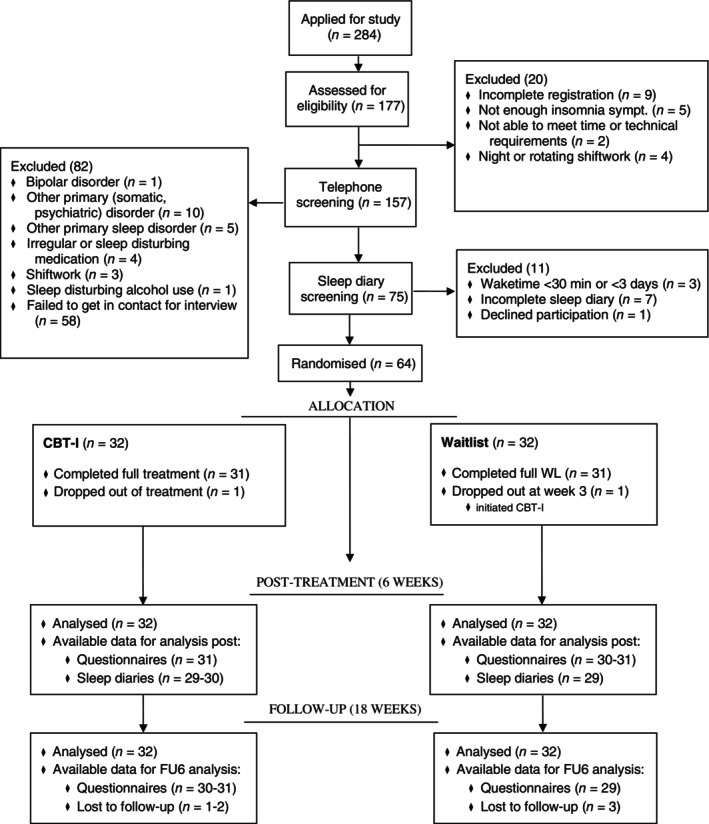
Flowchart. CBT‐I, cognitive behavioural therapy for insomnia; FU, follow‐up; sympt., symptoms; WL, waitlist.

Participants were recruited through advertisements in social media (Facebook, LinkedIn) from autumn 2021 to spring 2022. To be eligible, participants were asked to complete three screening phases, which consisted of a web questionnaire, a telephone interview, and a 7‐day sleep diary. Inclusion and exclusion for the three screening phases (see below for more detail) were based on expert recommendations for a standard research assessment of insomnia and The Diagnostic and Statistical Manual of Mental Disorders, Fifth Edition (Buysse et al., 2006; Diagnostic and Statistical Manual of Mental Disorders, 5th ed., 2013; Edinger et al., 2004; Lichstein et al., 2003). A summary of the flow of participants from signing up to the end of the trial is presented in Figure 1.

As a first step, candidates had to provide written consent, create an account on the study's internet platform, and complete a web‐based screening questionnaire, which was the first part of the three screening phases. In this first phase, the following inclusion criteria were required: sleeping difficulties ≥3 nights/week during at least 3 months despite adequate opportunities for sleep and an ISI score indicative of insomnia disorder (i.e., a total score of ≥11, a score of ≥2 on one of the three questions concerning night‐time symptoms (item 1–3), and a score of ≥2 on at least one of the two questions regarding daytime interference and distress (items 5 and 7) (Bastien et al., 2001; Morin et al., 2011). Candidates also needed to confirm that they were aged ≥18 years, had time and opportunity to participate in treatment, and would be able to perform homework assignments 30–60 min/week. Finally, candidates were also required to have access to a cellphone, mobile number, email address, and internet access. Severe depression (≥21 points on the 21‐item Depression, Anxiety and Stress Scale‐21 [DASS‐21]; Alfonsson et al., 2017) or suicidal ideation (≥4 points on item 9 of the Montgomery–Åsberg Depression Rating Scale – Self report [MADRS‐S]; Svanborg & Åsberg, 1994) was used as a precursor for further risk assessment in the telephone interview.

In the second phase, eligible candidates were screened with a 30‐min semi‐structured telephone interview based on the Duke Structured Interview for Sleep Disorders (DSISD) and the Mini‐International Neuropsychiatric Interview (MINI; Edinger et al., 2011; Sheehan et al., 1997) to assess sleep and mental disorders. The aim of the interview was to identify and exclude insomnia due to contextual conditions (e.g., pregnancy, and animals or disturbing sounds in the sleep environment), night or rotating shift work (more than three shifts changes a week), elevated intake of alcohol (more than two standard drinks a day) or caffeine (more than four beverages a day or more than two after 6:00 p.m.), or if the candidate had been administered CBT‐I within the past 5 years. In terms of comorbid conditions, they were, for inclusion, required to be stable and/or under relevant treatment regimes, and that insomnia was the most disabling and distressing condition or an issue despite treatment for the somatic or psychiatric comorbidity. The following criteria were employed for medication: (a) if sleep medication was reported, it had to be relatively fixed (e.g., in dosage) for the past 3 months, (b) if a selective serotonin reuptake inhibitor was used, the onset of the medication or the last change of dosage needed to be ≥3 months before the telephone interview, and (c) if participants were reporting sleep‐disturbing medication use, they were excluded. Candidates with a history of psychotic or bipolar disorders and those with other primary sleep disorders other than insomnia (sleep apnea, restless legs syndrome, periodic limb movement disorder, circadian rhythm disorder, and parasomnias) were excluded.

During the third screening phase, eligible candidates were asked to complete a 7‐day sleep diary (see assessment measures below for more information). For inclusion, the candidate was required to report ≥3 days of sleep difficulties in the diary, i.e., initiating, maintaining, or waking up too early, defined as ≥30 min for each occasion.

Participants registering sleep diaries were continuously scanned by the second author, and if sleep‐diary criteria were met for inclusion, the participants’ study identifiers (IDs) were sent to the first author for single randomisation (using an internet‐based random generator [www.randomizer.org] to one of the two groups [CBT‐I or WL]). A total of 64 participants with insomnia disorder met the study criteria CBT‐I (*n* = 32) and WL (*n* = 32). Participants allocated to CBT‐I were informed that they were to be contacted within 2 weeks for treatment initiation by their therapist. Those allocated to WL were informed that they were included in the group that was asked to register their sleep in a sleep diary for 5 weeks and answer five weekly questionnaires and a 3‐month follow‐up before receiving CBT‐I free of charge, as well as two extra vouchers of 300 SEK (Swedish Krona) each at the post‐treatment and follow‐up assessment for participating and waiting on receiving their treatment. Thus, there was no blinding of allocation.

### 2.3 Intervention

The CBT‐I treatment in this study was delivered through a smartphone app containing five weekly treatment modules, with a 15‐min telephone support call on six occasions.

The treatment content delivered through the smartphone app was structured as a self‐help format aimed at providing sufficient information for participants to implement the cognitive and behavioural techniques on their own. Each weekly module contained between three and 12 sections that structured the weekly content and consisted of short, animated video instructions that described and directed the participant on how to solve tasks inherent in each weekly module, such as performing and registering exercises, as well as making a sleep plan for the week ahead. The estimated time spent reviewing the mandatory three to 12 sections of each weekly module was ~10–15 min (not including the time spent implementing the material throughout the week). For a detailed outline of the weekly content of each module, see Table S1.

The cognitive and behavioural techniques included in this treatment consisted of sleep restriction (Spielman et al., 1987), stimulus control (Bootzin et al., 1991), sleep hygiene, cognitive restructuring, and relaxation, with a greater emphasis on sleep restriction and stimulus control throughout the 5 weeks (for a treatment outline over the 6 weeks, see Table S1 in supporting information). Sleep restriction (Kyle et al., 2015; Spielman et al., 1987) was initiated at the beginning of Week 2 in treatment and continuously calculated on the previous 7‐days of sleep diary, with a minimum sleep window of 5 h. A sleep efficiency of >85% was used as a criterion for increasing the sleep window by 15 min throughout the active phase (Weeks 2–5), and an efficiency of <80% either meant a reduction by 15 min or an unchanged sleep window. The positioning of the sleep window was based on the patient's daily schedule and type of sleep complaint. Napping was instructed to be avoided.

In conjunction with the material in the smartphone app, each participant was also offered a weekly 15‐min telephone support call. The five weekly calls, scheduled at the initiation of the treatment, were made by a therapist at the start of each week and delivered to follow‐up on the following week, providing feedback and troubleshooting any potential obstacles to proceeding with the protocol, and ended with a short presentation of the content and planning for the coming week. The sixth and final call at the end of treatment (beginning of Week 6) was aimed at summarising and ending the treatment and, if requested, booking a booster call at 1‐month post‐treatment. The two therapists (the second author and an employee at Learning to Sleep) delivering the call were licensed psychologists and experienced practitioners in providing CBT‐I. To minimise the risk of contamination and secure the integrity of the treatment, a therapist manual was developed, describing how to deliver the support call, the possible content to be brought up each week, and how to navigate potential questions out of topic. Contamination and integrity issues were also automatically handled by the fact that each participant received the same digitalised treatment and the support call concerning only the content in the app for each respective week. During treatment, both therapists met on a need‐to basis to discuss treatment and integrity issues. To proceed to the next week, the participant had to walk through each section of the preceding week or participate in the phone call to check they were still sufficiently on track. If problems with proceeding with the weekly call occurred during treatment, the therapist contacted the participant through the chat function in the smartphone app to reschedule or decide to skip the call depending on the need and the progress of the individual participant.

### 2.4 Assessment procedure

Assessment measures were administered at the following time points: diagnostic measures at pre‐treatment, treatment credibility and expectancy during the first week of therapy, night‐time symptoms, and primary outcome at pretreatment, and weekly from the start to end of treatment, and primary outcome also at the 3‐month follow‐up. Secondary outcomes were assessed at pre‐ and post‐treatment and at the 3‐month follow‐up, and sick‐leave, healthcare consumption, and concomitant insomnia treatment at post‐treatment and follow‐up. In addition, the participants in the CBT‐I group completed measures on their perception of treatment, therapists, treatment satisfaction, workload, their activity, and adverse events at posttreatment.

For the CBT‐I group, all self‐report measures were delivered via the smartphone app. Specifically, when a questionnaire was delivered, a push‐notification showed up on the smartphone, prompting the participant to answer. For the WL group, an email containing a weblink to the questionnaire was posted. In total, three email reminders were sent out if an assessment had not been completed.

### 2.5 Diagnostic measures

To evaluate diagnostic criteria for sleep disorders, the DSISD, a semi‐structured interview with good reliability and validity (Edinger et al., 2011) was used. To identify psychiatric comorbidities, the MINI version 6.0.0 (2009‐02‐20, Swedish version), a semi‐structured clinical interview, with demonstrated good reliability and validity (Sheehan et al., 1997) was used with the following modules: Major depressive disorder, Suicidality, Mania, Panic disorder, Social phobia, Post‐traumatic stress disorder, Psychotic disorder, and Generalised anxiety disorder.

Both interviews were conducted by the last author and a certified psychologist working on the project, and both interviewers received supervision on a need‐to basis from the first author.

### 2.6 Primary outcome

To measure participants’ global perception of insomnia severity the ISI (Bastien et al., 2001; Morin et al., 2011) was used. The ISI consists of seven items rated on a 5‐point scale (0–4) with a total score of 0–28 and assesses both daytime and night symptoms (e.g., daytime distress and functioning, noticeability due to sleep the problem, and night‐time difficulties with initiating and maintaining sleep) for the previous 2 weeks. To capture the relevant timeframe for the weekly assessments, the timeframe was changed to 1 week, for the weekly assessments. The ISI was further used to classify the number of responders and remitters at post‐treatment and follow‐up, where a response to treatment was defined as achieving a reduction of ≥8 points and remission as a score of <8 at post‐treatment (Morin et al., 2011). The ISI is a well‐established scale with adequate internal consistency (Cronbach's alpha = 0.91) and temporal stability (*r* = 0.80), as well as sensitivity to therapeutic changes (Morin et al., 2004, 2005, 2009). The scale has also been validated for use over the internet (Thorndike et al., 2011). In this sample, the internal consistency of the ISI was relatively low at α = 0.66.

### 2.7 Secondary outcomes

Nighttime symptoms were assessed by using the 7‐day Consensus Sleep Diary (Carney et al., 2012; Morin, 1993). The diary assessed bedtime, sleep onset, wakeup time, risetime, number of awakenings, and wake after sleep onset (WASO). Based on these assessments, the online diary calculated and displayed sleep onset latency (SOL) and sleep efficiency. For the analyses, we also computed early morning awakening (EMA), time in bed, total wake time, and total sleep time (TST). The sleep diary is considered the ‘gold standard’ subjective measure of sleep with reliable estimates of sleep (Buysse et al., 2006).

To measure the degree of functional impairment associated with insomnia, the Work and Social Adjustment Scale (WSAS) was administered (Mundt et al., 2002). The WSAS is a five‐item questionnaire rated on a 9‐point scale (0–8, total score of 0–40) that assesses functioning across work and home management, social and private leisure activities, and relationships with others. WSAS has previously showed robust psychometric properties (Jansson‐Fröjmark, 2013) and in the present sample demonstrated a Cronbach's alpha of 0.91.

To assess the level of depression, anxiety, and stress, the DASS‐21 (Alfonsson et al., 2017) was used. The DASS‐21 is a 21‐item questionnaire containing three subscales with seven items, each designed to assess depression, anxiety, and stress. The scale has demonstrated acceptable psychometric properties (Alfonsson et al., 2017), and in the present sample, Cronbach's alpha was 0.91 for the depression subscale, 0.71 for the anxiety factor, and 0.81 for the stress subscale.

To evaluate quality of life, the Brunnsviken Brief Quality of Life scale (BBQ; Lindner et al., 2016) was used. The BBQ aims to measure quality of life in six areas, using two items per area, resulting in a total of 12 items. The BBQ has shown good psychometric properties and sensitivity for discriminating non‐clinical and clinical samples, as well as response to treatment (Andersson et al., 2017; Lindner et al., 2016). The Cronbach's alpha was 0.83 for the BBQ in the present sample.

### 2.8 Treatment credibility, expectancy, and satisfaction

Participants’ perceived expectancy and credibility for the intervention were assessed by the Credibility/Expectancy Questionnaire (CEQ; Devilly & Borkovec, 2000). The CEQ is a six‐item questionnaire with acceptable psychometric properties and ability to predict outcomes (Borkovec & Costello, 1993; Devilly & Borkovec, 2000).

To assess participants’ satisfaction with their treatment, the eight‐item Client Satisfaction Questionnaire (CSQ‐8; Attkisson & Zwick, 1982) was used. The CSQ‐8 has demonstrated high internal consistency (Attkisson & Zwick, 1982).

### 2.9 Treatment dropout and adherence

To assess treatment attrition and activity, data were summarised regarding the number of support calls, minutes of telephone support, the number of treatment dropouts, modules, and the degree of total exercises completed.

### 2.10 Self‐rated experience of treatment, therapists, workload, and activity

To evaluate participants’ treatment experience, we administered self‐formulated questions assessing their perception of treatment, the therapist's support, and their own activity during treatment. Treatments were evaluated in terms of how interesting and relevant the content was and the amount of presented information. The therapist's support was evaluated by questions assessing how often issues were brought up with the therapists and the amount of help received. Finally, participants’ activity was assessed in terms of hours devoted to treatment per week (i.e., reading texts, watching animated content, and performing exercises), amount of the total material that participants made use of and implemented during treatment (%), and the frequency with which they worked on exercises.

### 2.11 Sick leave, healthcare consumption, concomitant insomnia treatment

To control for potential group differences in sick leave, healthcare consumption, and concomitant insomnia treatment, participants were assessed with self‐formulated questions at post‐treatment and at follow‐up on their potential occurrence. Regarding sick leave, participants were asked how many days they had been on sick leave using three response categories (0, 1–14, and 15–180 days). Healthcare consumption was assessed by asking whether participants had sought any healthcare for their sleeping problems during the past 5 weeks or 3 months using three response categories (‘no’, ‘yes: in regular care’, or ‘yes: outside regular care’). Finally, to assess whether there had been any concomitant insomnia treatment, participants were asked whether they had undergone any other insomnia treatments for their sleeping problems during the past 5 weeks or 3 months using three response categories (‘yes: with sleeping pills’, ‘yes: with other pharmacological agents’, or ‘yes: with non‐pharmacological treatments’ [i.e., psychological, and alternative treatments]).

### 2.12 Adverse events

At post‐treatment, participants were, based on previous research (Kyle et al., 2011), asked to rate if any of 14 common adverse events had occurred because of CBT‐I and whether any of these events had prevented activity in CBT‐I.

### 2.13 Data analytical plan and statistical analyses

Measurements related to baseline characteristics (e.g., age, education, medications), treatment activity, and experiences (e.g., CEQ, CSQ, treatment dropout, adverse effects, therapist support, and activity) were only analysed for descriptive purposes, using the mean or crosstab function in Statistical Package for the Social Sciences (SPSS), version 28.0.0.0 (190).

For the primary data analysis, full information maximum likelihood estimation with non‐normality robust standard errors was fitted for both the continuous and categorical outcomes, using ‘Mplus’ version 7.4. (Muthén & Muth́en, 2015). In line with the intention‐to‐treat principle, all available data from all randomised individuals were analysed. Full information maximum likelihood estimation is one of two recommended methods for handling missing data because it provides unbiased estimates and standard errors under a more lenient missing data assumption (i.e., missing at random) by modelling all available observations jointly in estimating model parameters without imputation (Enders, 2020; Schafer & Graham, 2002). The comparisons were two‐tailed and examined as statistically significant at *p* < 0.05, using normal theory tests (i.e., estimate/standard error [SE]). Confidence intervals (CIs) are provided with a 95% margin, and effect sizes for the continuous outcomes are derived from the model‐implied means at the two endpoint assessments and the standard deviation (SD) at baseline and presented as the standardised mean differences (*d*) (Feingold, 2009). Whereas for the categorical outcomes, the effect sizes are calculated based on the unstandardised beta coefficient from the regression models and presented as an odds ratio (OR) estimate.

To model individual change as a function of conditions and handle dependence in the data due to repeated‐measures over time, latent growth modelling with random effects (person‐specific trajectories) was applied as the primary analytical approach (Bollen & Curran, 2006). One of the main advantages of latent growth modelling is that it allows the possibility to model and capture change at both the group and individual levels as a continuous latent process over time instead of incremental change, which allows for better model fit to the data with smaller errors (Hesser, 2015). The model construction followed recommendations for building growth models (Bollen & Curran, 2006; Hesser, 2015) and was based on visual inspection of individual trajectories and observed means, as well as analytical assessment of model fit, using indices for growth models (Wu et al., 2009). Correlated subject‐specific random coefficients (i.e., random intercept and slopes) were retained, and error terms in the models were allowed to vary over time whenever they significantly contributed to the model. For all models, a binary variable representing the two conditions (WL as the reference category) was included as a fixed predictor of linear, quadratic, and or cubic trajectories in the models to examine average differential change as a function of condition (CBT‐I versus WL). Model fit for all models was considered acceptable using the Comparative Fit Index (CFI) and standardised root mean squared residual (SRMR) index (Bentler, 1990; Hu & Bentler, 1999), albeit two models, SOL and anxiety (CFI: 0.811–0.832, SRMR: 0.199), were borderline outside the acceptable range.

For the primary outcome measure the ISI, which was measured weekly during the active treatment period to the post‐treatment assessment, and at follow‐up ‐ a piecewise growth model (Bollen & Curran, 2006; Raudenbush & Bryk, 2002) was fitted to satisfactorily model and capture differential linear and non‐linear change as a function of treatment during the distinct phases of the trial (i.e., pre–post and follow‐up phase). For the first piece (weekly from pre‐ to post‐assessments), the trajectories were modelled with a random linear and quadratic slope, whereas trajectories during the second piece (post‐ and follow‐up assessments) were modelled as a fixed linear slope.

For the night‐time symptoms (SOL, WASO, EMA, TST) as assessed by the sleep diary and aggregated to six weekly mean time‐points from pre‐ to post‐treatment, four different growth models were fitted for each outcome. The fitted models ranged from applying a random linear slope to adding a fixed or random quadratic and cubic slope to fit the data properly and simultaneously handle improper solutions due to convergence issues in the models (Chen et al., 2001).

For the secondary outcomes, assessed at pre‐, post‐, and follow‐up, two growth models, one from pre‐ to post‐treatment and one from post‐treatment to follow‐up, were fitted for each outcome. The growth models were specified with a random intercept and a fixed slope to model change during the pre–post assessment and from post‐treatment to follow‐up.

Finally, the response and remission rates extracted from the primary outcome ISI (defined under measures) were analysed from pre‐ to post‐treatment and from pre‐treatment to the 3‐month follow‐up using logistic regression, with pre‐treatment scores on the ISI included to control for pretreatment variations.

## 3 RESULTS

### 3.1 Sample and patient characteristics

For the total sample (*n* = 64), the mean (SD) age was 47.2 (10.4) years, 76.6% (*n* = 49) were females, 25% (*n* = 16) reported use of sleeping pills, 12.5% (*n* = eight) stated a psychiatric and 34.4% (*n* = 22) a somatic comorbid disorder, and 45.3% (*n* = 29) used medications for somatic conditions. For more details on participants, see Table 1.

**TABLE 1:** Participant characteristics at pretreatment.

Characteristic	WL (*n* = 32)				CBT‐I (*n* = 32)			
	%	*N*	Mean	SD	%	*N*	Mean	SD
Sex (female)	81	26			72	23		
Age, years		32	48.4	11.8		32	45.9	8.7
Marital status								
Single	34.4	11			37.5	12		
Married/partner	65.6	21			62.5	20		
/separated								
Education								
Elementary school	3.1	1			3.1	1		
High school	37.5	12			12.5	4		
University	59.4	19			84.4	27		
Employment								
Employed	71.9	23			87.5	28		
Student	6.3	2			9.4	3		
Unemployed	3.1	1			3.1	1		
Other	18.8	6			0	0		
Insomnia duration, years			10.7	11.2			8.3	10
Insomnia severity index		32	18.6	4.4		32	19.2	3.3
Comorbidity								
Somatic^a^	37.5	12			31.3	10		
Psychiatric^b^	15.6	5			9.4	3		
Medication								
Sleep^c^	18.8	6			31.3	10		
Other^d^	56.3	18			34.4	11		

Abbreviations: CBT‐I, cognitive behavioural therapy for insomnia; ISI, Insomnia Severity Index; SD, standard deviation; WL, waitlist.

^a^ The following somatic conditions were reported (with number of participants in parenthesis): asthma or allergy (five), hypothyroidism (five), chronic pain or migraine (three), autoimmune diseases (four), hypertension (two), osteoporosis (one), blood disease (one), irritable bowel syndrome (one), chronic fatigue syndrome (one), reproductive system disease (one), and eye disease (one).

^b^ The following psychiatric conditions were diagnosed (with number of participants in parenthesis): depression (three), generalised anxiety disorder (two).

^c^ The following sleep medications were reported (with number of participants in parenthesis): anti‐histamines (10), hypnotics (five), and melatonin (four).

^d^ The following non‐sleep medications were reported (with number of participants in parenthesis): for hypertension or heart diseases (eight), for thyroid gland (seven), antidepressants (six), for asthma or allergy (four), anti‐inflammatory (four), for menopausal symptoms (three), bronchodilators (three), for urinary disease (one), for cancer (one), for migraine (one), for multiple sclerosis (one), and for osteoporosis (one).

### 3.2 Participants adherence, activity, and perception of treatment, workload, activity, and therapist‐support

Outlined in Table 2 are descriptive statistics on variables related to undergoing treatment, such as participants rated credibility in and expectancy for treatment before initiation, as well as how satisfied they were post‐treatment. Results showed that the CBT‐I was perceived as credible, that participants expected to benefit from undergoing CBT‐I, and that participants were satisfied with CBT‐I at post‐treatment. Furthermore, Table 2 outlines measures of participants’ treatment adherence, including the mean number of modules reached, exercise completion rates, number of support calls, and their length in minutes, as well as the number of participants receiving a booster session. In short, results indicate low attrition and good adherence (for further details, see Table 2). Finally, Table 2 also displays participants’ perception of CBT‐I, their therapist, the workload, and their own activity. In brief, the descriptive statistics in Table 2 demonstrates that the treatment was often interesting, balanced in the amount of information, that issues with implementing CBT‐I were often brought up with their therapist, and that they received a lot of help from their therapist on the issues raised. In terms of their self‐rated activity, participants rated that they spent around 1–2 h per week on the treatment, that they made use of close to 75% of the material, and that they often worked on the material (For further details, see Table 2).

**TABLE 2:** Treatment credibility, expectancy, satisfaction, activity, sleep medications, and self‐rated: Activity and user‐experience.

	Cognitive behavioural therapy for insomnia		
	(*N* = 32)		
	Mean (SD)	*N*	%
*CEQ*			
Credibility	19.7 (3.7)	30	
Expectancy	21.0 (3.8)	30	
*CSQ*	29.5 (2.9)	31	
*ACTIVITY MEASURES*			
Treatment dropout		1^a^	3.1
Number of modules reached^b^	5.9 (0.6)		
Module with all exercises completed (1–6)	4.1 (1.3)		
Number of support calls (1–6)^c^	5.9 (0.6)	188 (calls)	
Number of booster sessions	‐	22/30	73
Total minutes of support calls (1–6)	101.9 (14.4)		
Minutes per support calls^d^	17.3 (2.2)		
*PERCEPTION OF TREATMENT*			
Amount of information^e^	2.8 (0.4)	31	
Media perceived as interesting/relevant^f^	3.9 (0.8)	31	
How often issues with treatment were brought up with the therapist^g^	3.96 (1.4)	31	
Degree of help received from therapist with issues brought up^h^	4.7 (0.6)	31	
*SELF‐RATED ACTIVITY*:			
Hours/week spent on the treatment^i^			
0–1 h	1.8 (0.6)	9	29
1–2 h		20	64.5
3–4 h		2	6.5
Amount of information digested and implemented^j^	4.2 (0.7)	31	
Degree of work invested in exercises^k^	3.6 (0.9)	31	
*SLEEP MEDICATION PRACTICE*:			
Use of medications at start of treatment		8	25
Discontinued during treatment		7	87.5

Abbreviations: CEQ, Client Expectancy Questionnaire; CSQ, Client Satisfaction Questionnaire; SD, standard deviation.

^a^ Dropped out at module three.

^b^ Represents the mean number of modules reached, as a measure of adherence.

^c^ All participants finalised all six calls except two (one dropped out of treatment at module three, one missed call three).

^d^ Represents the weekly mean for all participants. The following response alternatives were used for:

^e^ 1–5 (‘way too little’, ‘too little’, ‘ok’, ‘too much’, ‘way too much’).

^f^ 1–5 (‘never’, ‘rarely’, ‘sometimes’, ‘often’, ‘always’).

^g^ 0–5 (‘never’, ‘rarely’, ‘sometimes’, ‘often’, ‘always’).

^h^ 1–5 (‘no help’, ‘a little help’, ‘some help’, ‘to a huge degree’, ‘much help’).

^i^ 1–5 (0–1 h, 1–2 h, 3–4 h, 4–5 h, >5 h).

^j^ 1–5 (0%, 25%, 50%, 75%, 100%).

^k^ 1–5 (‘not at all’, ‘rarely’, ‘now and then’, ‘often’, ‘very often’).

### 3.3 Analysis of treatment effects

#### 3.3.1 Primary outcome

Table 3 depicts the observed weekly ISI measurements for both groups and the estimated differential effect between the WL and CBT‐I groups during active treatment and follow‐up phases, respectively, as well as the estimated mean difference at post‐treatment and follow‐up. As observed in Table 3, both the CBT‐I and WL groups improved, although the CBT‐I group had a steeper slope, which resulted in a final larger improvement. This was also reflected in the estimate for the differential effect for CBT‐I, where the term that tested differential linear change from pre‐ to post‐treatment was significant. It is worth noting that the observed differential significant effect in favour of CBT‐I was in comparison to a significant reduction on the ISI in the WL group from pre‐ to post‐treatment.

**TABLE 3:** Observed means and results from piecewise growth models examining change over the pre–post assessment and 3‐month follow‐up for the primary outcome.

Outcome	Observed means				Results from linear growth models				
	WL		CBT‐I		Effect of predictor on linear and quadratic slope			Group difference at POST and FU	
	*N*	Mean (SD)	*N*	Mean (SD)	Predictor	Estimate (SE)	*p*	Mean difference (95%CI)	Effect size
ISI score									
PRE	32	18.6 (4.4)	32	19.2 (3.3)					
W1	31	16.2 (4.8)	31	15.5 (4.5)					
W2	31	14.9 (5.0)	31	12.5 (4.4)					
W3	30	14.8 (5.8)	31	9.4 (3.9)					
W4	29	15.0 (5.9)	29	8.1 (4.9)	linear 1	−1.778 (0.715)	0.013*		
POST	31	14.7 (5.4)	31	6.0 (4.9)	quadr 1	−0.029 (0.126)	0.818	−8.894 (−11.33, −6.46)	−2.261
FU	29	14.2 (6.3)	31	7.5 (5.5)	linear 2	0.196 (0.086)	0.023*	−6.545 (−9.49, −3.60)	−1.664

*Note*: The growth model is based on available data for the intention‐to‐treat sample (*n* = 62). Treatment conditions were included as fixed binary coded predictors using the control condition as the reference category. The estimates are the unstandardised regression coefficients for the group differences across the active treatment phase (linear 1, quadr 1) and from post‐treatment to follow‐up (linear 2) and can be interpreted as an effect size in the original metric of the scale (one‐time unit is 1 week). The unstandardised mean difference (unstandardized effect size) and effect size (standardised mean difference) were derived from the model estimates. The negative mean difference at post‐treatment and at follow‐up indicates a beneficial effect for CBT‐I relative to WL on ISI.

Abbreviations: CBT‐I, cognitive behavioural therapy for insomnia; FU, follow‐up; ISI, Insomnia Severity Index; PRE, pre‐treatment; POST, post‐treatment; quadr 1, refers to the estimation of quadratic time across pre‐ to post‐treatment; SD, standard deviation; SE, standard error; linear 1 and linear 2, the estimation of linear time across pre‐ to post‐treatment, and post‐treatment to follow‐up; W, Week; WL, waitlist.

* Values statistically significant at *p* < 0.05.

From post‐treatment to follow‐up, as also depicted in Table 3, the terms that tested differential change for CBT‐I indicated a close to zero deterioration (estimate = 0.196), which, however, was significant. Worth mentioning here is that this was a test of differential effect to a WL that continued to improve during the same period. Together, this indicates a sustained effect during the follow‐up period for the WL group and a minor deterioration compared to an improved WL for the CBT‐I group. Observed in Table 3 are also the associated effect sizes, derived from the model‐implied means, indicating a large effect at post‐treatment (*d* = 2.26) and large but attenuated at follow‐up (*d* = 1.66) compared to the WL group.

**FIGURE 2:**
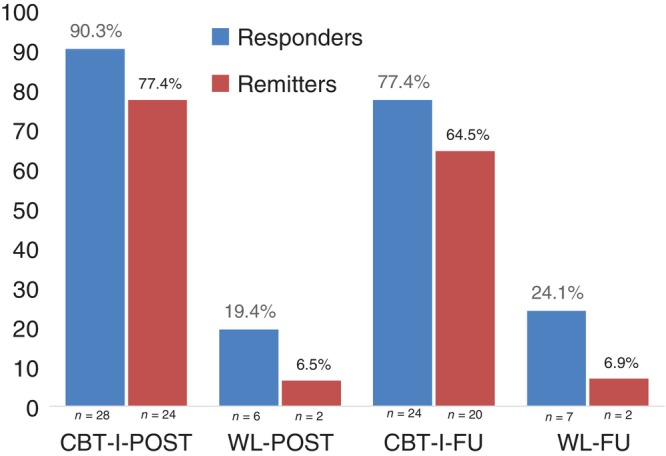
Percentage response and remission based on the Insomnia Severity Index scores (observed means). CBT‐I, cognitive behavioural therapy for insomnia; FU, follow‐up; POST, post‐treatment; WL, waitlist.

Finally, as depicted in Figure 2, are the observed proportion of responders and remitters according to the ISI thresholds at post‐treatment and follow‐up. As can be observed, the rate of response and remission was large in comparison to the WL, and this was also reflected in the logistic regressions. In terms of response, there was a statistically significant difference between the CBT‐I and the WL groups at post‐treatment (estimate [SE] = 3.75 [0.83], *p* < 0.05) and at follow‐up (estimate [SE] = 2.43 [0.64], *p* < 0.05) with associated OR effect sizes of 42.66 and 11.36, respectively. For remission, there was also a statistically significant difference between the CBT‐I and WL groups at post‐treatment (estimate [SE] = 4.41 [1.04], *p* < 0.05) and at follow‐up (estimate [SE] = 3.73 [1.1], *p* < 0.05) with associated OR effect sizes of 82.36 and 41.49, respectively.

#### 3.3.2 Secondary outcomes

Table 4 provides the results from the estimated growth models for the four night‐time symptoms assessed from the sleep diary, along with associated model‐implied unstandardised and standardised mean differences (*d*) at Week 5 (endpoint of active treatment phase).

**TABLE 4:** Observed means and results from the growth models examining change over the pre‐post assessment for the secondary nighttime symptoms.

Outcome	Observed means				Results from linear growth models				
	WL			CBT‐I	Effect of predictor on linear and quadratic slope			Group difference at POST	
	*N*	M (SD)	*N*	M (SD)	Predictor	Estimate (SE)	*p*	Mean difference (95% CI)	Effect size
SOL									
PRE	32	76.2 (66.4)	32	53.6 (42.1)					
W1	30	54.7 (35.6)	32	47.8 (33.4)					
W2	31	61.8 (47.2)	31	30.6 (25.0)					
W3	31	65.2 (67.8)	32	23.3 (14.4)	linear	−17.05 (12.3)	0.166		
W4	30	68.6 (68.8)	31	23.6 (18.8)	quadr	−13.91 (6.3)	0.028*		
POST	29	53.7 (40.6)	29	19.5 (9.2)	cubic	2.02 (0.8)	0.017*	−31.97 (−46.5, −17.5)	−0.658
WASO									
PRE	32	44.1 (28.5)	32	49.8 (38.2)					
W1	30	46.8 (34.1)	32	37.9 (25.8)					
W2	31	39.2 (26.2)	31	23.4 (18.3)					
W3	31	34.2 (24.1)	32	18.6 (15.5)					
W4	30	39.8 (33.6)	31	18.8 (19.6)	linear	−10.64 (3.7)	0.004*		
POST	29	35.6 (26.3)	30	14.5 (11.3)	quadr	1.23 (0.61)	0.042*	−18.90 (−29.22, −8.52)	−0.602
EMA									
PRE	32	46.4 (29.5)	32	41.7 (33.6)					
W1	30	36.7 (16.07)	32	41.0 (30.6)					
W2	31	37.1 (29.9)	31	26.2 (22.0)					
W3	31	36.3 (23.9)	32	22.9 (20.7)					
W4	30	38.8 (24.0)	31	19.0 (14.7)					
POST	29	35.6 (20.8)	29	20.6 (17.1)	linear	−3.49 (1.29)	0.007*	−19.62 (−28.9, −10.3)	−0.681
TST									
PRE	32	348.5 (55.0)	32	368.6 (66.7)					
W1	30	373.0 (64.5)	32	377.9 (59.0)					
W2	31	379.3 (59.7)	31	337.3 (62.0)					
W3	31	383.8 (64.6)	32	350.8 (64.4)	linear	−45.37 (14.3)	0.002*		
W4	30	380.3 (74.3)	31	369.6 (48.9)	quadr	11.10 (7.2)	0.124		
POST	29	386.3 (55.2)	29	382.4 (52.3)	cubic	−0.59 (0.97)	0.540	−1.999 (−24.1, 28.1)	−0.031

*Note*: The growth model is based on available data for the intention‐to‐treat sample (*n* = 62). Treatment conditions were included as fixed binary coded predictors using the control condition as the reference category. The estimates are the unstandardised regression coefficients for the group differences across the active treatment phase (linear, quadr, cubic) and can be interpreted as an effect size in the original metric: minutes (one‐time unit is 1 week). The unstandardised mean difference (unstandardised effect size) and effect size (standardised mean difference) were derived from the model estimates. The negative mean difference at post‐treatment and at follow‐up indicates a beneficial effect for CBT‐I relative to WL on the Insomnia Severity Index (except for TST).

Abbreviations: CBT‐I, cognitive behavioural therapy for insomnia; EMA, early morning awakening; linear, quadr, and cubic, refers to the estimation of linear, quadratic, and cubic time from pre‐ to post‐treatment; PRE, pre‐treatment; POST, post‐treatment; SD, standard deviation; SE, standard error; SOL, sleep onset latency; TST, total sleep time; WASO, wake after sleep onset; W, Week; WL, waitlist.

* Values statistically significant at *p* < 0.05.

For SOL, a cubic model was fitted due to the change in direction at two time points during the active treatment period for the WL group. As can be observed in Table 4, the quadratic and the cubic terms in the model that tested the difference between the CBT‐I and WL groups between pre‐ and post‐assessment were statistically significant. This reflected a steeper improvement of the CBT‐I group from Week 1 to Week 4 and the reverse, although of a much lower magnitude (cubic term) compared to the quadratic term, from Week 4 to Week 5. In all, this resulted in a significant endpoint difference in favour of the CBT‐I group.

For WASO, a quadratic model was fitted, where both the linear and the quadratic term that tested the difference between the two groups were significant, indicating a steeper reduction for the CBT‐I group that subsequently changed direction (quadratic, *p* < 0.05) and flattened out compared to the WL group. Together, this resulted in a significant endpoint difference in favour of the CBT‐I group.

For EMA, a linear model was fitted, where the term that tested the differential effect of CBT‐I was significant. This reflected a steeper slope (decrease) compared to the WL group, which also resulted in a significant endpoint difference in favour of the CBT‐I group.

Finally, for TST, a cubic model was fitted, where both the linear and the quadratic term that tested the difference between the two groups was significant. This reflected a decrease for the CBT‐I group at Week 2, which subsequently changed direction and resulted in a non‐significant endpoint difference between the two groups.

All effects, except for TST, favoured the therapies over the WL, and estimates of between‐group effect sizes were in the medium range for the difference between The CBT‐I and the WL groups at Week 5 (range *d* = 0.65–0.72). For TST, the growth curve showed a reduction in TST during treatment that returned to the pretreatment value and, at Week 5, was non‐significant from the WL group and of low effect (*d* = 0.031).

Table 5 depicts the observed means for the pre‐, post‐treatment, and follow‐up measurements assessing functional impairment, depression, anxiety, stress, and quality of life, as well as the estimates from the two latent growth models for each outcome, including the differential linear change over pre‐ to post‐treatment and post‐treatment to follow‐up, along with model‐implied mean differences at post‐treatment and follow‐up. The term in the growth model that tested differential linear change over the treatment phase was significant for the WSAS and DASS‐21: Stress, indicating a larger reduction in favour of the CBT‐I group.

**TABLE 5:** Observed means and results from the linear growth model examining change over the pre–post, and post‐follow‐up assessment for the secondary outcomes.

Outcome	Observed means				Results from linear growth models				
	WL		CBT‐I		Effect of predictor on linear and quadratic slope			Group diff at POST and FU	
	*N*	Mean (SD)	*N*	Mean (SD)	Predictor	Estimate (SE)	*p*	Mean diff (95%CI)	Effect size
WSAS									
PRE	32	20.3 (9.4)	32	19.0 (9.3)					
POST	30	17.7 (9.6)	31	10.1 (9.2)	linear 1	−6.62 (2.24)	0.003*	−7.97 (−12.55, −3.38)	−0.87
FU	29	14.6 (8.9)	31	10.0 (8.9)	linear 2	3.19 (1.70)	0.061	−4.56 (−9.04, −0.08)	−0.50
BBQ									
PRE	32	52.8 (20.7)	32	56.8 (20.2)					
POST	30	57.9 (22.3)	31	68.1 (21.8)	linear 1	6.58 (4.01)	0.101	10.61 (−0.05, 21.27)	0.51
FU	29	58.1 (22.3)	30	64.2 (22.7)	linear 2	−3.84 (3.29)	0.243	6.87 (−4.09, 17.84)	0.32
DASS‐21									
Depression									
PRE	32	4.9 (4.7)	32	3.8 (3.7)					
POST	30	4.3 (4.2)	31	2.3 (3.1)	linear 1	−0.99 (0.63)	0.116	−2.12 (−3.89, −0.35)	−0.54
FU	29	3.5 (3.8)	30	4.1 (4.4)	linear 2	2.143 (0.67)	0.001*	0.18 (−1.95, 2.30)	0.05
Anxiety									
PRE	32	3.3 (3.0)	32	2.4 (2.6)					
POST	30	2.5 (2.7)	31	0.9 (1.0)	linear 1	−0.84 (0.60)	0.165	−1.71 (−2.71, −0.72)	−0.7
FU	29	2.3 (2.9)	30	1.5 (1.9)	linear 2	0.73 (0.53)	0.167	−0.98 (−2.20, 0.26)	−0.44
Stress									
PRE	32	7.7 (4.1)	32	6.9 (4.0)					
POST	30	6.8 (4.2)	31	4.1 (3.0)	linear 1	−2.15 (0.85)	0.011*	−2.93 (−4.70, −1.16)	−0.77
FU	29	6.0 (3.7)	30	4.9 (3.4)	linear 2	1.31 (0.80)	0.102	−1.43 (−3.21, 0.37)	−0.40

*Note*: The growth model is based on available data for the intention‐to‐treat sample (*n* = 62). Treatment conditions were included as fixed binary coded predictors using the control condition as the reference category. The estimates are the unstandardised regression coefficients for the group differences, across the active treatment phase (linear 1), and from post‐treatment to follow‐up (linear 2) and can be interpreted as an effect size in the original metric of the scale (one‐time unit is 1 week). The unstandardised mean difference (unstandardised effect size) and effect size (standardised mean difference) were derived from the model estimates. The negative mean difference at post‐treatment and at follow‐up indicate a beneficial effect for CBT‐I relative to WL on the Insomnia Severity Index.

Abbreviations: BBQ, Brunnsviken Brief Quality of Life Scale; CBT‐I, cognitive behavioural therapy for insomnia; DASS‐21, 21‐item Depression, Anxiety and Stress Scale; FU, follow‐up; linear 1 and linear 2, estimation of linear time across pre‐ to post‐treatment, and post‐treatment to follow‐up; PRE, pre‐treatment; POST, post‐treatment; SD, standard deviation; SE, standard error; W, Week; WSAS, Work and Social Adjustment Scale; WL, waitlist.

* Values statistically significant at *p* < 0.05.

Besides the differential linear change, there were at post‐treatment also significant endpoint differences in favour of CBT‐I on the WSAS and DASS‐21: Stress, anxiety, and depression. The estimated endpoint between‐group mean difference effect sizes at post‐treatment were all medium to large (range *d* = 0.51–0.87).

Finally, in the follow‐up models, the terms that tested differential linear change over the follow‐up phase as a function of treatment were significant for depression, indicating that the CBT‐I group deteriorated, while the WL improved from post‐treatment to follow‐up, and the rest of the outcomes at follow‐up were maintained during the follow‐up phase.

The WSAS was the only endpoint difference that was significant at follow‐up, thus maintaining a significant endpoint difference from post‐treatment, although smaller. At follow‐up, the estimated endpoint between‐group mean difference effect sizes were all small to medium (range *d* = 0.05–0.50).

#### 3.3.3 Sick leave, healthcare consumption, and concomitant insomnia treatment

As illustrated in supplemental Table S2, no significant difference was found between the groups at post‐treatment or at follow‐up, although the number of other treatments received at follow‐up approached significance (χ^2^[2] = 3.210, *p* = 0.072), indicating that participants in the WL group might have undergone other treatment to a higher degree at follow‐up compared to the CBT‐I group (for a more detailed description of these results, see supplemental Table S2).

#### 3.3.4 Adverse events

After CBT‐I, 25 of 31 participants reported experiencing one or more adverse events. However, only eight of 31 reported difficulties with following through with CBT‐I due to the adverse event, with fatigue/exhaustion being experienced as both the most treatment‐disturbing event and the most frequently reported event by 23 of 31 participants. The range of reported events per participant went from zero to nine, with a mean of four reported events per participant (100 events/25 participants). For more details, see Table S3 in the supplemental materials.

## 4 DISCUSSION

The purpose of this trial was to investigate the clinical efficacy of smartphone app‐delivered CBT‐I relative to a sleep diary WL control. To the best of our knowledge, this is the first trial examining CBT‐I delivered exclusively via a smartphone app with telephone support on a rigorously screened sample with insomnia disorder. The findings from the study support the hypothesis of a significantly larger effect for the app‐delivered CBT‐I on insomnia severity and also showed that CBT‐I resulted in significantly stronger improvements in the majority of the secondary outcomes.

In comparison with meta‐analytical findings for CBT‐I relative to passive comparators, the improvements in the present trial were substantially higher on the ISI (*d* = 2.26 versus *g* = 0.98) but relatively similar on SOL (*d* = 0.66 versus *g* = 0.57), WASO (*d* = 0.60 versus *g* = 0.63), and TST (*d* = 0.03 versus *g* = 0.16) (Straten et al., 2018). However, one caveat should be underscored in this comparison; while the present study's CBT‐I was based on modern treatment principles, primarily with a focus on sleep restriction and stimulus control as core strategies, CBT‐I in the meta‐analysis consisted of a wide spectrum of interventions, i.e., relaxation, sleep restriction, paradoxical intention, identifying and challenging unhelpful beliefs about sleep, and various combinations of the interventions (Baglioni et al., 2019; Riemann et al., 2023). Other factors, such as patient characteristics, therapist support, and homework compliance, should also be mentioned as potentially vital when comparing effects across trials and meta‐analyses. The more potent improvement in the ISI in this study could be due to the CBT‐I approach itself (smartphone‐delivered core treatment strategies with therapist support), but it could also be since ISI improvements appear to be associated with feeling less tired and anxious after CBT‐I, and not sleep improvements per se (Marway et al., 2023). The remaining outcomes in this trial have previously not been meta‐analysed comparing CBT‐I with passive comparators, but earlier data support the notion that the present study's CBT‐I was more or similarly effective than other CBT‐I approaches on the BBQ, DASS, and WSAS relative to passive comparators (Espie et al., 2014; Sunnhed et al., 2019).

There are also additional findings from the present trial worth reflecting upon, particularly because most trials on CBT‐I do not assess treatment‐related factors and as the results support the continued use of the CBT‐I approach examined in this study. Overall, CBT‐I was perceived as credible, interesting, and well‐balanced, with high expectancy and high satisfaction, associated with low attrition and high adherence, and with high ratings of the content, exercises, and support from a therapist. Several of these treatment‐related factors have been poorly studied in the context of CBT‐I due to factors such as a lack of well‐defined approaches to assess the factors, a few published trials, and issues with small sample sizes (Agnew et al., 2021; Condon et al., 2021; Matthews et al., 2013). The above findings are thus difficult to compare with extant research, which relates particularly to adherence and adverse events. Despite knowledge gaps and methodological limitations, it is, however, safe to state that the dropout rate from this study was low (0% dropped out from the trial, 3.1% dropped out of the treatment). Dropping out from internet‐delivered CBT‐I trials has been estimated at 24.7%, and from face‐to‐face delivered CBT‐I trials, between 0.0% to 33% (Okajima et al., 2010; Zachariae et al., 2016). Future research is important concerning treatment‐related factors for CBT‐I, particularly when smartphone‐delivered CBT‐I will (hopefully) be compared against active and placebo conditions. In such studies, adherence, adverse events, attrition, and other treatment‐related factors can be monitored during the whole study period to investigate potential differences across treatment modalities.

Given the novelty of telephone support as applied in this trial, it is worth reflecting on its potential implications. Although the design in this study cannot delineate causality, participants rated that they brought up issues and received help to a large degree from their therapist, potentially explaining the high adherence, low dropout, and large effect sizes in this trial; results that are in line with previous research on therapeutic guidance (Baumeister et al., 2014; Hasan et al., 2021). However, although there are different ways to implement guidance, with fully automated versions (Espie et al., 2012; Morin, 2020), asynchronous text messages (Blom et al., 2024), guidance on demand, or telephone support by an educated sleep expert that in this trial yielded promising outcomes—human guidance also introduces barriers for scaling the intervention. For this trial specifically, there is a barrier in the limited sleep experts available and the support each of them can provide. Nevertheless, the mean of 17 min support a week in this trial still allows for more treatments per therapist than face‐to‐face treatments. Thus, while therapist telephone support hinders scalability, it also seems to aid the individuals in troubleshooting, adhering, and proceeding through difficult parts of treatment. In this way, therapist telephone support might circumvent dropout and potential negative consequences of treatment failures (e.g., reduced likelihood of seeking further treatment), as well as optimise outcomes through greater adherence, similar to how health professionals monitor treatments in regular healthcare (Blom et al., 2024). Although this trial indicates favourable outcomes using weekly telephone support by a psychologist, the question of what constitutes optimal guidance for securing scalable interventions with low dropout, high adherence, and large effects is still open. Future research should, therefore, investigate by whom (e.g., therapist, research assistant, automated), through what modality (e.g., text, telephone, video), for whom (e.g., level of insomnia severity, comorbidities), at what stage in treatment, with what frequency (bi‐weekly, on‐demand), characterises the optimal guidance for fully implementing the active treatment ingredients, reach significant effects, and avoid dropouts.

There are also some additional strengths and limitations that need to be highlighted. Obvious strengths of the present trial are that the participants being administered smartphone‐delivered CBT‐I were guided by a therapist, that the sample was rigorously screened, that the study allowed medicated and comorbid patients to participate to a large extent, that attempts were made to estimate the clinical efficacy of CBT‐I on a broad range of insomnia‐relevant domains, and that several treatment‐related areas (e.g., support, adherence, satisfaction, and dropout) were assessed. Several methodological limitations should also be noted. First, the CBT‐I smartphone app was compared with a WL condition, which consisted only of completing sleep diaries for 6 weeks. As a result, the efficacy of the CBT‐I smartphone app is still unknown relative to more potent comparisons, e.g., face‐to‐face CBT‐I, placebo conditions, and pharmacotherapy. Second, although the screening in this trial was a methodological strength, applying three steps of screening might also pose a barrier to scalability. On the other hand, securing the correct diagnosis requires a rigorous assessment, as is also emphasised by modern guidelines (Riemann et al., 2023). Third, although the app format of this trial contains several advantages in terms of reducing treatment barriers, the optimal format for patients with insomnia is still an open question. Fourth, the efficacy of the CBT‐I smartphone app was only examined up to 3 months after therapy, so the long‐term effect is not known beyond that time point. However, in relation to previous trials, a notable strength is that the efficacy of CBT‐I at follow‐up was controlled. Fifth, all the study participants were recruited through social media advertisements. As self‐referred participants are likely to differ from patients in regular care (Davidson et al., 2009), the generalisability of the present findings is unknown. The generalisability of the sample might also be questioned due to the sociodemographic profile of the sample; e.g., the fact that most of the participants were female and highly educated makes the generalisability less certain. Also, some of the mean scores on the pretreatment self‐report scales indicate that the present sample was less affected by other problems than insomnia, relative to previous studies; e.g., the mean scores on the BBQ and WSAS indicate better quality of life and functioning (Jansson‐Fröjmark & Jacobson, 2021). Sixth, although the assessment of efficacy included several domains, other areas, e.g., cost‐effectiveness and fatigue, were not measured as outcomes. Seventh, although there were written guidelines and continuing discussion of how the screening and telephone guidance should be carried out, the study did not employ a monitoring system for how this clinical work was executed; as a result, the way screening and guidance were delivered could differ across participants. Eight, Cronbach's alpha for the ISI was relatively low at 0.66, implying some uncertainty regarding the one‐dimensionality of the ISI and the interpretation of the results on the ISI.

Although this study and previous trials demonstrate promising findings for smartphone‐delivered CBT‐I, more research is still needed to further our knowledge about unknown aspects. In particular, future research is warranted to assess the app's efficacy relative to more potent comparisons (e.g., face‐to‐face CBT‐I), to other delivery formats (e.g., through a computer, by video link), and its long‐term effects. Also, more research is needed to examine the generalisability of the app, e.g., by including patients with insomnia comorbid with another mental disorder and recruiting participants from regular care settings. It is also vital for future research to explore the efficacy of smartphone‐delivered CBT‐I on other key outcomes, such as cost‐effectiveness, and using a monitoring system for screening and treatment to further ensure that all study participants receive the same procedure. Finally, there is also a need for continued research comparing various formats of delivering CBT‐I: face‐to‐face in individual or group formats, internet administration, and bibliotherapy versus smartphone delivery.

The present clinical trial demonstrates that smartphone‐delivered CBT‐I with therapist support has the potential to be an effective and acceptable intervention for patients with insomnia disorder. As there are still unknown areas concerning digital forms of CBT‐I, we recommend more research into smartphone‐delivered and other digitalised forms of CBT‐I. To reduce the burden of insomnia disorder, we also urge the healthcare sector across the globe to implement validated, smartphone‐delivered CBT‐I with therapist support in healthcare settings.

## AUTHOR CONTRIBUTIONS

**Markus Jansson‐Fröjmark:** Validation; supervision; conceptualization; methodology; writing – review and editing. **Rikard Sunnhed:** Writing – original draft; writing – review and editing; funding acquisition; data curation; formal analysis; project administration; supervision; conceptualization; methodology.

## CONFLICT OF INTEREST STATEMENT

Dr Markus Jansson‐Fröjmark has nothing to disclose. Learning to sleep has funded 15% over 6 months of Dr Rikard Sunnhed's employment for planning and operation of the clinical trial, as well as providing telephone support for participants in the treatment arm.

## Supporting information


**Data S1:** Supplementary Information.

## Data Availability

The data underlying this article cannot be shared publicly since it has not been approved by the participants.
