# Supplementary material for: Smartphone application‐delivered cognitive behavioural therapy for insomnia with telephone support for insomnia disorder compared to a waitlist control: a randomised clinical trial
Source: J Sleep Res. 2024 Oct 8;34(3):e14363. doi: 10.1111/jsr.14363 (PMC12069742; doi:10.1111/jsr.14363)
Supplement: Supplementary file 1 — Data S1: Supplementary Information. [file JSR-34-e14363-s001.docx]

*Supplemental material*

S1, Supplemental Table 1. *Treatment Outline*

*Note.* The table outlines a conceptual overview of the treatment content of each weekly app-module. The weekly telephone-support call (at the beginning of each week), besides offering feedback and troubleshooting on the app content, also assessed sleep medication use and offered guidance and support in discontinuing use of sleep medications.

S2, Supplemental Table 2. *Participants’ sick leave, healthcare consumption and other insomnia treatments assessed at post treatment and at 6-month follow-up.*

*Note.* Based on observed data, n=31 for CBT at post and follow up, and n=30 at post and n=29 at follow up for the waitlist. CBT = Cognitive Behavior Therapy, WL = waitlist. ^a^ The most common treatment reported for all groups at posttreatment was use of sleep hypnotics (4/5) followed by other pharmacological agents (1/5). At follow-up, the most common treatment reported for all groups was the use of sleep hypnotics (5/11), followed by other pharmacological agents (4/11), followed by over-the-counter pills (2/11).

*S3,* Supplemental Table 3. *Adverse events for the treatment group assessed at post treatment.*

*Note.* Based on observed data, n=31.

Supplemental Table 1. *CBT-I Outline*

|  | Interventions and homework assignments in the Learning to sleep smartphone-app of Cognitive Behavioral Therapy for Insomnia |
| --- | --- |
| 1 | - Treatment introduction (info about sleep, sleep problems and CBT) - Individualized case conceptualization - Sleep diary and worksheet registration (throughout the treatment) - Psychoeducation: CBT and sleep - 3 exercises: My sleep, my thoughts (about sleep) and my treatment goals |
| 2 | - Psychoeducation: Sleep restriction and stimulus Control - Assessing values from diary - Sleep restriction: Implementation - Stimulus Control: Implementation - Exercise: Sleep restriction and stimulus Control |
| 3 | - Sleep restriction and stimulus control: Adjustment, adherence - Problem-solving and strategies for maintaining sleep restriction and stimulus control. - Psychoeducation: Sleep hygiene - routines and rules. - Exercise: routines and rules |
| 4 | - Sleep restriction and stimulus control: Adjustment, adherence and problem solving. - Psychoeducation: Cognitive strategies (unhelpful beliefs and negative thoughts) and relaxation exercises - 3 exercises: worry time; play the script till the end; relaxation image |
| 5 | - Sleep restriction and stimulus control: Adjustment, adherence and problem solving. - Explore progress: problem solve if needed. - Psychoeducation: Maintenance and plan for the future - Exercise: what interventions and components to maintain; personal plan for the future |
| 6 | - Exploring progress and goal attainment - Perpetuate routines and goals. - Revision of case conceptualization and plan for the future |
| 7 | - Booster session (1 month from session 6) - Exploring progress and goal attainment - Relapse prevention and treatment consolidation |

*Note.* The weekly telephone-support call (at the beginning of each week), besides offering feedback and troubleshooting on the app content, also assessed sleep medication use and offered guidance and support in discontinuing use of sleep medications.

Supplemental Table 2. *Participants’ sick leave, healthcare consumption and other insomnia treatments assessed at post treatment and at 6-month follow-up.*

|  | All groups | | CBT | | WL | |  |
| --- | --- | --- | --- | --- | --- | --- | --- |
|  | *%* | *n* | *%* | *n* | *%* | *n* | χ^2^ |
| Sick leave |  |  |  |  |  |  |  |
| POST | 16.4 | 10 | 16.1 | 5 | 16.7 | 5 | 0.003, p = 0.613 |
| FU6 | 30 | 18 | 29.0 | 9 | 31.0 | 9 | 0.029, p = 0.544 |
| Sought health care |  |  |  |  |  |  |  |
| POST | 1.6 | 1 | 0 | 0 | 3.3 | 1 | 1.051, p = 0.492 |
| FU6 | 5 | 3 | 0 | 0 | 10.3 | 3 | 3.376, p = 0.107 |
| Received other treatments ^a^ |  |  |  |  |  |  |  |
| POST | 8.2 | 5 | 3.2 | 1 | 13.3 | 4 | 2.070, p = 0.167 |
| FU6 | 18.3 | 11 | 9.7 | 3 | 27.6 | 8 | 3.210, p = 0.072 |

*Note.* Based on observed data, n=31 for CBT at post and follow up, and n=30 at post and n=29 at follow up for the waitlist. CBT = Cognitive Behavior Therapy, WL = waitlist. ^a^ The most common treatment reported for all groups at posttreatment was use of sleep hypnotics (4/5) followed by other pharmacological agents (1/5). At follow-up, the most common treatment reported for all groups was

the use of sleep hypnotics (5/11), followed by other pharmacological agents (4/11), followed by over-the-counter pills (2/11).

Supplemental Table 3. *Adverse events for the treatment group assessed at post treatment.*

|  | *Adverse events not hindering treatment* | |  | *Events hindering treatment* | |
| --- | --- | --- | --- | --- | --- |
|  | *n* | *%* |  | *n* | *%* |
| Participants experiencing (n) | 25 | 80.6 |  | 8 | 25.8 |
| Frequency of reported events: |  |  |  |  |  |
| Low mood | 8 | 25.8 |  | 1 | 3.2 |
| Fatigue/exhaustion | 23 | 74.2 |  | 6 | 19.4 |
| Extreme sleepiness | 18 | 58 |  | 5 | 16.1 |
| Feeling agitated | 6 | 19.3 |  | 1 | 3.2 |
| Bodily pain | 2 | 6.4 |  | 1 | 3.2 |
| Headache/migraine | 5 | 16.1 |  | 1 | 3.2 |
| Euphoria | 3 | 9.7 |  | 1 | 3.2 |
| Reduced motivation/energy | 13 | 41.9 |  | 1 | 3.2 |
| Changes in hunger/appetite | 5 | 16.1 |  | 1 | 3.2 |
| Blurred vision | 4 | 12.9 |  | 1 | 3.2 |
| Dizziness | 2 | 6.4 |  | 1 | 3.2 |
| Feeling irritable | 11 | 35.5 |  | 1 | 3.2 |

*Note.* Based on observed data, n=31.

.
